# Epidemiology, classification, treatment, and mortality of adult femoral neck and basicervical fractures: an observational study of 40,049 fractures from the Swedish Fracture Register

**DOI:** 10.1186/s13018-021-02701-1

**Published:** 2021-09-15

**Authors:** Jonas Sundkvist, Anders Brüggeman, Arkan Sayed-Noor, Michael Möller, Olof Wolf, Sebastian Mukka

**Affiliations:** 1 Department of Surgical and Perioperative Sciences, Orthopaedics, Umeå University, Umeå, Sweden; 2 Section of Orthopaedics, Department of Surgical Sciences, Uppsala University, Uppsala, Sweden; 3 Department of Clinical Science and Education, Karolinska Institutet, Södersjukhuset, Stockholm, Sweden; 4 Institute of Clinical Sciences, Sahlgrenska Academy, Gothenburg University, Gothenburg, Sweden

## Abstract

**Background:**

Although femoral neck fractures (FNFs) are common in orthopedic departments, optimal treatment methods remain in dispute. There are few large nationwide studies, including basicervical FNFs (bFNFs), on epidemiology, treatment, and mortality. This nationwide study aims to describe the epidemiology, fracture classification, current treatment regimens, and mortality of undisplaced and minimally displaced (Garden I–II, uFNF), displaced (Garden III–IV, dFNF) and bFNFs in adults.

**Methods:**

All FNFs, including bFNFs with a registered injury date between 1 April 2012 and 31 December 2020, were included in this observational study from the Swedish Fracture Register (SFR). Data on age, sex, injury mechanism, fracture classification, primary treatment, and seasonal variation were analyzed.

**Results:**

Some 40,049 FNFs were registered in the SFR. The mean age of the patients in the register was 80.3 (SD 11) years and 63.8% (25,567) were female. Of all FNFs, 25.0% (10,033) were uFNFs, 63.4% (25,383) dFNFs, and 11.6% (4,633) bFNFs. Non-surgical treatment was performed in 0.6% (261) of the patients. Internal fixation (IF) (84.7%) was the main treatment for uFNFs and arthroplasty (87.3%) for dFNFs. For bFNFs, IF (43.8%) and hip arthroplasty (45.9%) were performed equally often. Of the 33,105 patients with a 1-year follow-up mortality at 1-year was 20.6% for uFNF, 24.3% for dFNF, and 25.4% for bFNF.

**Conclusion:**

The main treatment of uFNFs is IF with screws or pins. Hip arthroplasty is the predominant treatment for dFNF. bFNF are more common than previously reported and treated with IF or arthroplasty, depending on patient age. These results may help health care providers, researchers and clinicians better understand the panorama of FNFs in Sweden.

**Level of Evidence:**

IV, retrospective cohort study.

## Introduction

Femoral neck fractures (FNFs) are a subset of proximal femoral fractures commonly encountered in orthopedic practice with significant morbidity and mortality [1]. FNFs are mainly classified into undisplaced or minimally displaced (Garden 1–2, uFNF) and displaced fractures (Garden 3–4, dFNF). A third, less studied category of fractures is the basicervical FNFs (bFNFs), defined as fractures through the base of the femoral neck at their junction with the intertrochanteric region [2].

The treatment of FNFs in patients > 60 years is still under debate [3–5]. The treatment of uFNFs has primarily consisted of internal fixation (IF). However, recently, hip arthroplasty has been proposed as a viable option to reduce reoperation rates and possibly improve functional outcome [3, 6]. The type of arthroplasty to be used—hemi- or total hip arthroplasty in the treatment of dFNF in elderly patients remains controversial [7]. The incidence and treatment of bFNFs has been found to vary notably [8–10].

This study describes the injury mechanism, fracture classification, sex and age distribution, seasonal variation, and primary treatment in patients with fracture along the anatomical femoral neck using the Swedish Fracture Register (SFR).

## Materials and methods

### Study design and setting

This observational register study was designed based on data derived from the SFR.

The SFR, established in 2011, is a national quality register for the management of fractures and treatment. Detailed data on patient and fracture characteristics, injury mechanism, and fracture treatment are recorded in each affiliated department via a pre-specified digital form by the treating physician. Only patients with a permanent Swedish personal identification number and fractures that have occurred in Sweden are registered. In the SFR, fractures are mainly classified according to the AO/OTA classification system. Several studies have found the registration in the SFR to have high accuracy and validity [11]. The proportion of departments affiliated with the SFR has increased gradually; in January 2014, 40% of affiliated departments were active. As of 1 January 2021, all orthopedic departments (*n* = 54) in Sweden are engaged in the SFR, i.e., 100% coverage. More than 500,000 fractures had been registered by the end of 2020.

The registration of FNFs in the SFR includes uFNFs (Garden 1–2, AO/OTA 31-B1), dFNFs (Garden 3–4, AO/OTA 31-B3) and bFNFs (AO/OTA 31-B2). Information is available on peri-implant and periprosthetic fractures (UCS classification) and open fractures based on the Gustilo-Anderson classification. The injury mechanism includes information on stress, spontaneous and pathological fractures. Treatment is registered with the chosen type of therapy (non-operative or operative). Operative treatments consist of fracture fixation, including types of osteosynthesis (screws or pins, sliding hip device (SHD), long and short intramedullary nails (IMNs), anatomic plates), arthroplasty (hemi- or total, cemented or cementless fixation), or other (i.e., excision arthroplasty).

### Patient selection

All non-pathological FNFs (ICD code S72.00/S72.01) in adults registered in the SFR between 1 April 2012 and 31 December 2020 were included. We included bilateral FNFs and excluded peri-implant, periprosthetic, and pathological fractures.

### Study variables

Epidemiological data on age, sex, injury date, injury mechanism and type (high or low energy) trauma, fracture classification (type, side, open/closed fracture), treatment, and mortality were analyzed. The injury mechanism was categorized as a simple fall, an unspecified fall, a transportation accident or any other cause. Primary treatment was studied in the following groups: IF with screws or pins, IMNs, SHD or hip arthroplasty (hemi- or total), or other (i.e., excision arthroplasty).

### Statistics

Variables are presented as the proportion of all fractures (%), i.e., the available number of inputs in the register excluding any missing values.

Nominal variables are presented as proportions of all fractures and scale variables as means ± standard deviation (±SD). An independent sample *t* test was used to compare scale variables. For the log-rank test *p* < 0.05 was considered significant.

Data analysis was performed with R statistical software, version 4.0.4.

### Ethics

The study was approved by the Swedish Ethical Review Authority (dnr: 2020-05439) and carried out according to the Helsinki Declaration.

## Results

### Study patients and descriptive data

**Fig. 1:**
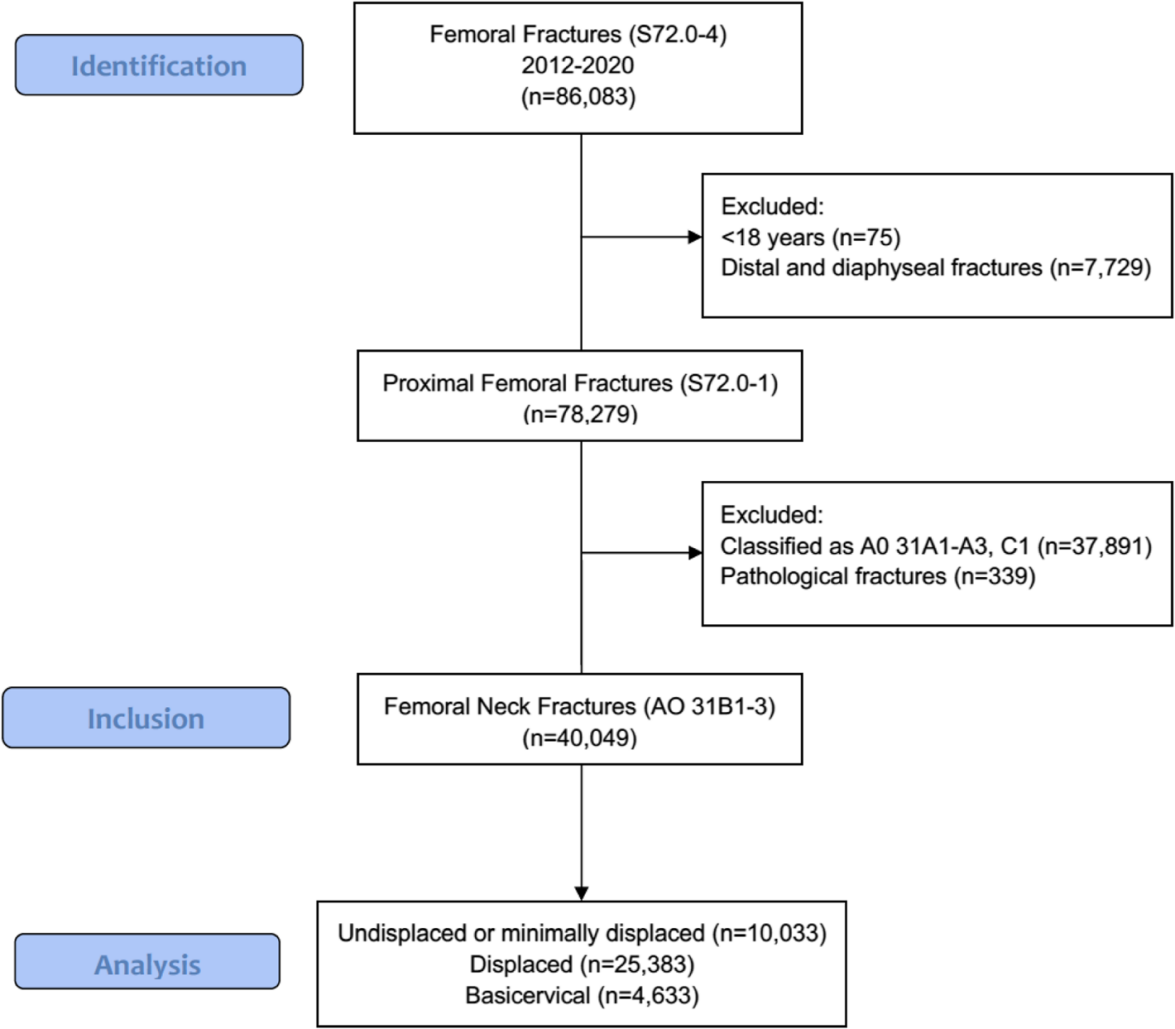
Flow diagram of patients

**Fig. 2:**
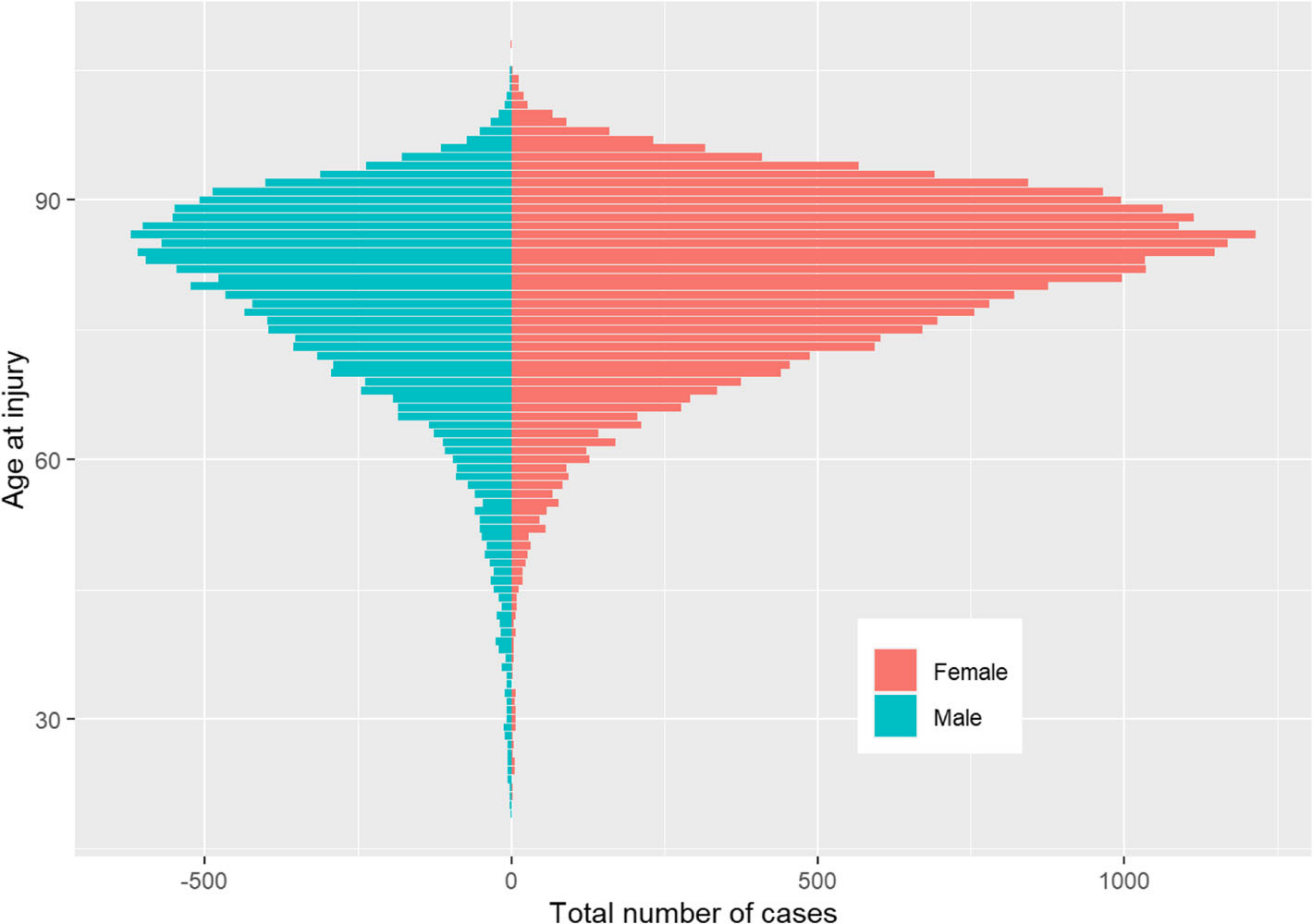
Distribution of age at injury

In total, 86,083 proximal femoral fractures were extracted from the SFR (ICD-10 S72.0-4). After exclusion, 40,049 FNFs were included for further analysis (Fig. 1). Some 3.7% (*n* = 1474) of the patients sustained bilateral FNFs during the study period. Of all fractures, 63.8% occurred in females (Table 1, Fig. 2). Females (mean age 81.3 years, SD ± 10) were older than men (78.7 years, SD ± 12, *p* < 0.001). Simple falls were the most common injury mechanism (79.9%). A minority of fractures were caused by high-energy trauma: 11.0% of the patients < 60 years of age and 0.6% > 60 years (Table 2). Some 5.8% were stress fractures in patients < 60 years and 1.1% in patients > 60 years. Most fractures occurred in the patients’ immediate environment (own home 50.1% or institution 14.4%).

**Table 1:** Patient characteristics. Distribution of sex, age, injury mechanism, type of trauma, and location

	Undisplaced or minimally displaced FNF	Displaced FNF	Basicervical FNF	All patients
	(***n*** = 10,033)	(***n*** = 25,383)	(***n*** = 4633)	(***n*** = 40,049)
Age ^*^	78.5 (± 12.0)	81.1(± 10.4)	80.2 (± 12.0)	80.3 (± 11.0)
Female ^†^	6,614 (65.9%)	16,290 (64.2%)	2663 (57.5%)	25,567 (63.8%)
Mechanism ^†^				
Bicycle^†^	92 (2.0%)	481 (1.9%)	92 (2.0%)	872 (2.2%)
Fall from height	288 (2.9%)	687 (2.7%)	128 (2.8%)	1103 (2.8%)
Fall same level	7857 (78.3%)	20,521 (80.8%)	3639 (78.5%)	32,017 (79.9%)
Stress fracture ^†^	190 (1.9%)	277 (1.1%)	74 (1.6%)	541 (1.4%)
Other cause ^‡^	474 (4.7%)	1003 (4.0%)	247 (5.3%)	1724 (4.3%)
Unspecified fall ^†^	925 (9.2%)	2414 (9.5%)	453 (9.8%)	3792 (9.5%)
Type of trauma				
High energy	117 (1.2%)	252(1.0%)	75(1.6%)	444 (1.1%)
Low energy	8827 (88.0%)	22,569(88.9%)	3,963(85.5%)	35,359 (88.3%)
Not applicable^‡^	190 (1.9%)	275(1.1%)	74 (1.6%)	539 (1.3%)
Unknown	183 (1.8%)	626(2.5%)	143(3.1%)	952 (2.4%)
Missing	716 (7.1%)	1,661(6.5%)	378(8.2%)	2755 (6.9%)
Location				
Home	4679 (46.6%)	13,000 (51.2%)	2389 (51.6%)	20,068 (50.1%)
Institution	1334 (13.3%)	33,794 (14.9%)	644 (13.9%)	5772 (14.4%)
Public place	409 (4.1%)	939 (3.7%)	167 (3.6%)	1515 (3.8%)
Street	538 (5.4%)	1218 (4.8%)	188 (4.1%)	1944 (4.9%)
Other	1137 (11.3%)	2006 (7.9%)	455 (9.8%)	3598 (9.0%)
Unspecified ^†^	1936 (19.3%)	4426 (17.4%)	790 (17.1%)	7152 (17.9%)

^*^The values are given as the mean and standard deviation

^†^Values are given as the number of patients, with the percent in parentheses

^‡^i.e., stress fracture

**Table 2:** Patient characteristics: distribution of sex, age at injury, injury mechanism, type of trauma and location stratified for patients ≤ 60 years of age at injury and those > 60 years

	Undisplaced or minimally displaced FNF		Displaced FNF		Basicervical FNF		All fractures	
	<= 60 (***N*** = 791)	> 60 (***N*** = 9242)	<=60 (***N*** = 1028)	> 60 (***N*** = 24,355)	<= 60 (***N*** = 307)	> 60 (***N*** = 4326)	<= 60 (***N*** = 2126)	> 60 (***N*** = 37,923)
**Age** (mean, SD)	50.6 (9.31)	80.9 (8.73)	51.8 (8.44)	82.4 (8.42)	49.8 (9.69)	82.3 (8.72)	51.1 (8.99)	82.0 (8.56)
**Sex**								
Female	388 (49.1%)	6226 (67.4%)	461 (44.8%)	15,829 (65.0%)	133 (43.3%)	2530 (58.5%)	982 (46.2%)	24,585 (64.8%)
**Side**								
Left	401 (50.7%)	4649 (50.3%)	553 (53.8%)	12,952 (53.2%)	152 (49.5%)	2184 (50.5%)	1106 (52.0%)	19,785 (52.2%)
**Mechanism**								
Fall same level	465 (58.8%)	7392 (80.0%	630 (61.3%)	19,891 (81.7%)	163 (53.1%)	3476 (80.4%)	1258 (59.2%)	30,759 (81.1%)
Fall from height	46 (5.8%)	242 (2.6%)	61 (5.9%)	626 (2.6%)	20 (6.5%)	108 (2.5%)	127 (6.0%)	976 (2.6%)
Unspecified fall	45 (5.7%)	880 (9.5%)	83 (8.1%)	2,331 (9.6%)	23 (7.5%)	430 (9.9%)	151 (7.1%)	3641 (9.6%)
Bicycle	95 (12.0%)	204 (2.2%)	90 (8.8%)	391 (1.6%)	30 (9.8%)	62 (1.4%)	215 (10.1%)	657 (1.7%)
Stress fracture	60 (7.6%)	130 (1.4%)	30 (2.9%)	247 (1.0%)	34 (11.1%)	40 (0.9%)	124 (5.8%)	417 (1.1%)
Other cause	80 (10.1%)	394 (4.3%)	134 (13.0%)	869 (3.6%)	37 (12.1%)	210 (4.9%)	251 (11.8%)	1473 (3.9%)
**Type of trauma**								
Low energy	552 (69.8%)	8275 (89.5%)	759 (73.8%)	21,810 (89.6%)	191 (62.2%)	3772 (87.2%)	1502 (70.6%)	33,857 (89.3%)
High energy	65 (8.2%)	52 (0.6%)	127 (12.4%)	125 (0.5%)	41 (13.4%)	34 (0.8%)	233 (11.0%)	211 (0.6%)
Not applicable	60 (7.6%)	130 (1.4%)	30 (2.9%)	245 (1.0%)	34 (11.1%)	40 (0.9%)	124 (5.8%)	415 (1.1%)
Unknown	42 (5.3%)	141 (1.5%)	46 (4.5%)	580 (2.4%)	18 (5.9%)	125 (2.9%)	106 (5.0%)	846 (2.2%)
Missing	72 (9.1%)	644 (7.0%)	66 (6.4%)	1595 (6.5%)	23 (7.5%)	355 (8.2%)	161 (7.6%)	2594 (6.8%)

### Seasonal variation

Both women and men appeared to sustain fewer fractures in temperate months (47.9%, April to September), and there was an observed slight increase in the colder months (52.1%, October to March).

### Fracture classification

Of all FNFs, 25.0% (10,033) were uFNFs, 63.4% (25,383) dFNFs, and 11.6% (4633) bFNFs.

In all three fracture types, men represented a majority in the age group < 60 years, whereas women represented the majority in the age group > 60 years (Table 2).

### Treatment

The method of surgical treatment differed between age groups and type of FNF (Table 3): patients < 60 years were mostly treated with IF for all types of FNF (74.3%).

**Table 3:** Treatment choice for the three fracture subgroups and overall fractures stratified by patients ≤60 years of age at injury and those > 60 years

	Undisplaced or minimally displaced FNF		Displaced FNF		Basicervical FNF		All fractures	
	<= 60 (***N*** = 791)	> 60 (***N*** = 9242)	<= 60 (***N*** = 1028)	> 60 (***N*** = 24,355)	<= 60 (***N*** = 307)	> 60 (***N*** = 4326)	<= 60 (***N*** = 2126)	> 60 (***N*** = 37,923)
Hip Screws	671 (84.8%)	7,582 (82.0%)	599 (58.3%)	1,554 (6.4%)	80 (26.1%)	302 (7.0%)	1,350 (63.5%)	9438 (24.9%)
Sliding hip device	37 (4.7%)	170 (1.8%)	38 (3.7%)	86 (0.4%)	138 (45.0%)	1237 (28.6%)	213 (10.0%)	1493 (3.9%)
Intramedullary nailing	2 (0.3%)	30 (0.3%)	4 (0.4%)	23 (0.1%)	12 (3.9%)	263 (6.1%)	18 (0.8%)	316 (0.8%)
Hemiarthroplasty cemented	0 (0%)	632 (6.8%)	60 (5.8%)	15,513 (63.7%)	8 (2.6%)	1455 (33.6%)	68 (3.2%)	17,600 (46.4%)
Hemiarthroplasty uncemented	0 (0%)	7 (0.1%)	2 (0.2%)	211 (0.9%)	1 (0.3%)	31 (0.7%)	3 (0.1%)	249 (0.7%)
Total hip arthroplasty cemented	7 (0.9%)	232 (2.5%)	208 (20.2%)	5621 (23.1%)	19 (6.2%)	543 (12.6%)	234 (11.0%)	6396 (16.9%)
Total hip arthroplasty uncemented	3 (0.4%)	4 (0.0%)	35 (3.4%)	86 (0.4%)	6 (2.0%)	15 (0.3%)	44 (2.1%)	105 (0.3%)
Total hip arthroplasty hybrid	1 (0.1%)	4 (0.0%)	29 (2.8%)	215 (0.9%)	5 (1.6%)	19 (0.4%)	35 (1.6%)	238 (0.6%)
Arthroplasty other	2 (0.3%)	6 (0.1%)	5 (0.5%)	147 (0.6%)	1 (0.3%)	20 (0.5%)	8 (0.4%)	173 (0.5%)
Non-operative	19 (2.4%)	101 (1.1%)	5 (0.5%)	94 (0.4%)	5 (1.6%)	24 (0.6%)	29 (1.4%)	219 (0.6%)
Other/unknown	49 (6.2%)	474 (5.1%)	43 (4.2%)	805 (3.3%)	32 (10.4%)	417 (9.6%)	124 (5.8%)	1696 (4.5%)

Patients > 60 years with uFNFs were largely treated with IF (84.2%), and to a much lesser extent, with hip arthroplasty (9.6%). Most patients > 60 years with a dFNF were treated with hip arthroplasty (89.4%), whereas IF was uncommon (6.8%).

Some 74.9% of the patients with bFNFs who were < 60 years of age were treated with IF (45.0% SHD) and 13% in this age group were treated with hip arthroplasty. Some 48.2% of the patients > 60 years with a bFNFs received a hip arthroplasty and 41.6% were treated with IF (7.0% screws or pins, 28.6% SHD).

### Mortality

**Fig. 3:**
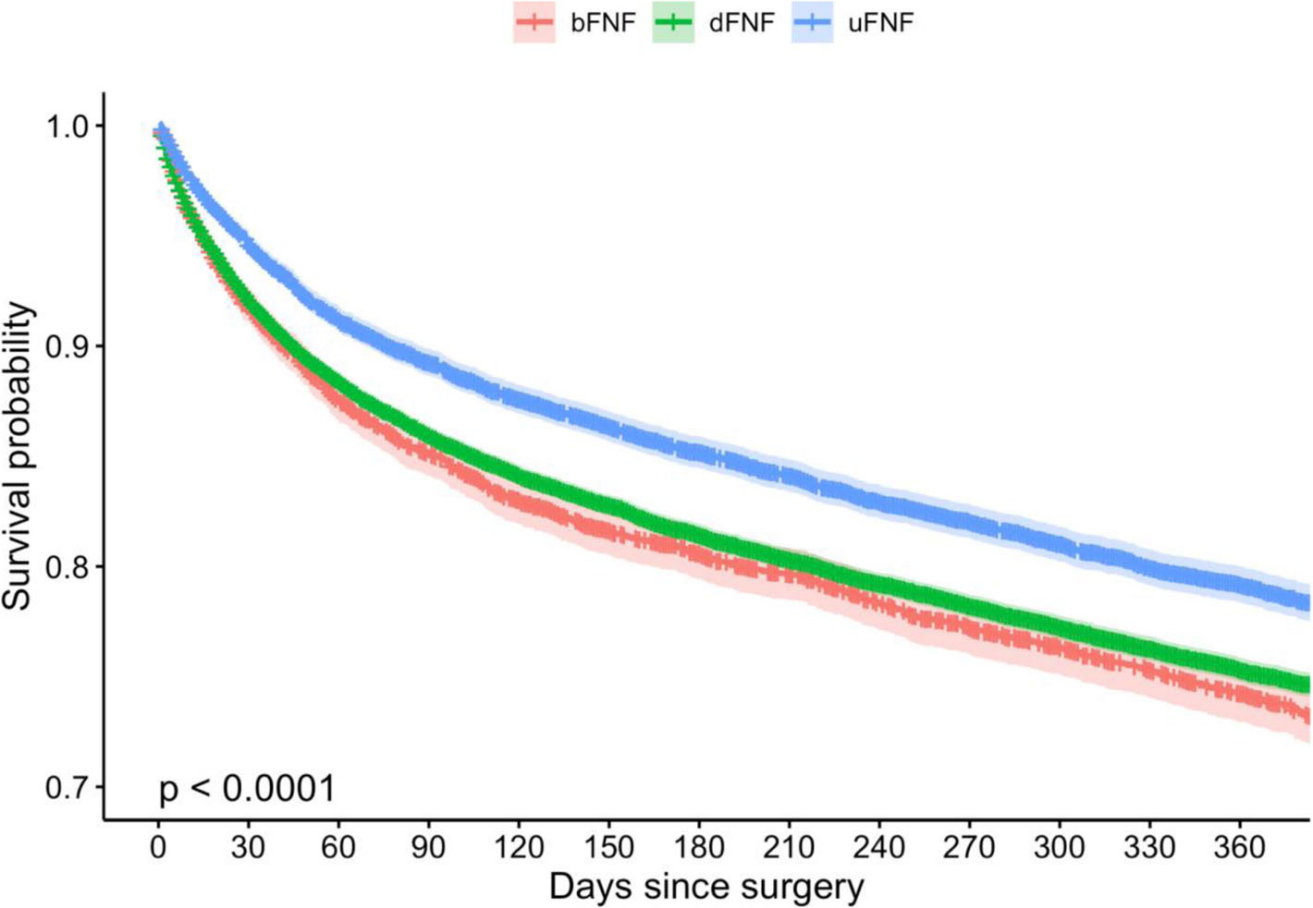
Survival function estimated by the Kaplan-Meier with mortality as endpoint

Of the 33,105 patients with a 1-year follow-up mortality for uFNF at 7-, 30-day, and 1-year mortality was 5.3%, 10.5%, and 20.6%. For dFNF 7-, 30-day, and 1-year mortality was 7.7%, 13.6%, and 24.3%. For bFNF 7-, 30-day, and 1-year mortality was 7.9%, 15.7%, and 25.4%. Kaplan-Meier curves are presented in Fig. 3.

## Discussion

The main findings of this study on data from the SFR are that the proportion of bFNFs is larger than previously described. The treatment varies between fracture groups but also depends on age. More men than women patients < 60 years old sustained a fracture caused by high-energy trauma or stress fractures.

### Demographics

As previously reported, more women than men sustain FNFs and females were older than men when the fracture occurs [1]. Most of the men were in the age group ≤ 60 years, for which high-energy trauma is more common, representing 11% of all fractures. In line with a previous study, the incidence of FNFs increases gradually with age, with a marked increase after age 75 [12]. Stress fractures accounted for 5.8% in the younger age group and have previously been reported mainly among military personnel in similar numbers [13]. These fractures are of interest due to their different background and typically delayed diagnosis with continued activity and risk of further displacement and worse long-term outcome [14]. Early diagnosis of stress fractures is essential to avoid serious complications. Persistent groin pain following exercise is a symptom worthy of attention and warrants thorough radiographic examination [15].

### Seasonal variation

The incidence of all fracture types varied over the calendar year. A slightly higher occurrence of FNFs was seen during the winter months (October–March) in both men and women. These results are supported by a report on seasonal variation of hip fractures [16]. Most hip fractures occur due to indoor falls and not slipping on icy roads or pavements, but the incidence has been proposed to increase with latitude and vary with season [17]. Vitamin D insufficiency has been suggested to influence fracture pathogenesis, particularly in northern climates [16]. The limited daylight in the northern latitudes during the winter months can potentially increase the risk of falls. There are contradictory reports of latitude on the risk of hip fracture incidence [18].

### Fracture classification

The classification of femoral fractures in the SFR is substantial (AO/OTA group) to almost perfect (AO/OTA type) and as accurate as in previous studies [11]. The SFR and AO/OTA currently do not support further classification into subgroups, i.e., include fracture tilt in the classification of uFNFs or the degree of displacement of bFNFs [19].

Previously, studies have used similar definitions of the bFNFs: a fracture medial to the intertrochanteric line [2]. However, these studies also included radiographic illustrations of fractures that extend laterally and distally into the trochanteric region and thus not according to the definition by AO/OTA [20, 21]. The AO/OTA definition of bFNFs is an extra-articular intracapsular fracture medial to the intertrochanteric line. Rotational instability is caused by the lack of muscular attachments to the proximal fragment. Hence, this group of fractures shares characteristics with uFNF and dFNF [22]. No differentiation is made between undisplaced and displaced fractures. The literature contains data with a wide range of incidence rates and treatment options, which could partly explain the varying definitions of these fractures [2]. In the present study, a large number of observers with a wide range of experience from junior doctors to senior consultants performed the classification in a clinical setting using the AO/OTA definition of bFNFs.

### Treatment

The type of surgical treatment depends on fracture localization, displacement, age, activity level, comorbidities, and surgeons’ preferences and practice patterns. The use of non-surgical treatment is uncommon in our study of all types of fractures.

The leading treatment of uFNFs is closed reduction (if necessary, to reduce tilt) and IF with either 2–3 cancellous screws or pins. Of those > 60 years, roughly 10% receive a primary hip arthroplasty, similar to a recent study from the Norwegian Hip Fracture Register [23]. These hip arthroplasties could be the clinical implication of fracture tilt on the lateral radiograph combined with those patients with coexisting degenerative joint disease or arthritic changes and a subsequent FNF [24–26]. The increased risk of treatment failure raises the question of whether hip arthroplasty has an advantage over IF [27]. There are no national guidelines in Sweden on how to treat uFNFs and whether to consider fracture tilt. A large nationwide register-based RCT has recently started in Sweden to study the benefit of hip arthroplasty in patients presenting with a uFNF [28].

Treatment of dFNFs in patients > 60 years mainly consists of hemi- or total hip arthroplasty, with the majority receiving a hemiarthroplasty through a direct lateral approach (approximately two thirds of all patients) [29]. The decision on the type of arthroplasty is contingent on many factors, including surgeon comfort and the patient’s age, health, and ambulatory status. In the present study, total hip arthroplasty was used in every fourth patient > 60 years; although a high proportion, it does correspond to that found in other Western countries [30–33]. This effect could be a patient-driven phenomenon due to an increasingly active elderly population with higher functional demands. However, benefits to the elderly with a total hip arthroplasty over hemiarthroplasty remain in dispute with similar short-term results [34, 35].

The operative management of bFNFs is more diverse. Most patients < 60 years are treated with IF and only a few have undergone arthroplasty. However, treatment for patients > 60 years is almost evenly divided between IF and arthroplasty. Commonly used implants are screws or pins, SHD and, to a lesser extent, intramedullary nails. Several observational cohort studies have not found superiority of one over the other for the treatment of bFNFs [8, 9, 36]. However, biomechanical studies demonstrate that screws or pins have a lower load to failure than SHD or intramedullary nails [37]. Hip arthroplasty has been less studied. Observational data suggest similar functional outcomes in comparison to IF [38]. Future studies are warranted to refine the definition, prognosis and type of surgical treatment to improve the outcome of bFNFs. The degree of fracture displacement along with the type of fracture, age, level of activity, and comorbidities influences the surgeon’s decision of treatment modality. Displacement, however, is not accounted for in the AO/OTA classification and cannot be analyzed in an observational study like ours.

### Mortality

Overall mortality was similar to previous reports on FNF [3, 6]. Although dFNF and bFNF had higher mortality than uFNF the previous groups were also older which makes comparison between the groups difficult.

### Strength and limitations

Given that we used the SFR with 100% coverage at the end of the study period to describe the epidemiology of FNFs, our study has the advantage of a large sample size. However, we are unable to identify the overall incidence given the stepwise introduction and the present completeness of the SFR in Sweden.

Because of the varied definitions of bFNFs in the literature, comparison to our results is limited [2]. In our study, the widely used AO/OTA definition of bFNFs strengthens the generalizability of our findings. The treating physicians and orthopedic surgeons performed the registrations and classification of fractures in the SFR. Of note, validation studies in various segments found the classification systems to be as accurate as previous validation studies of femoral fracture classification [11].

## Conclusion

The main treatment of uFNFs is IF with screws or pins, whereas hip arthroplasty is the primary treatment of dFNFs. bFNFs are more common than previously reported and treatment is more diverse and evenly distributed between IF and arthroplasty. Age and fracture type are factors affecting treatment choice. These results may help health care providers and clinicians better understand the panorama of FNFs in Sweden.

## Figures and Tables

**Fig. 4:**
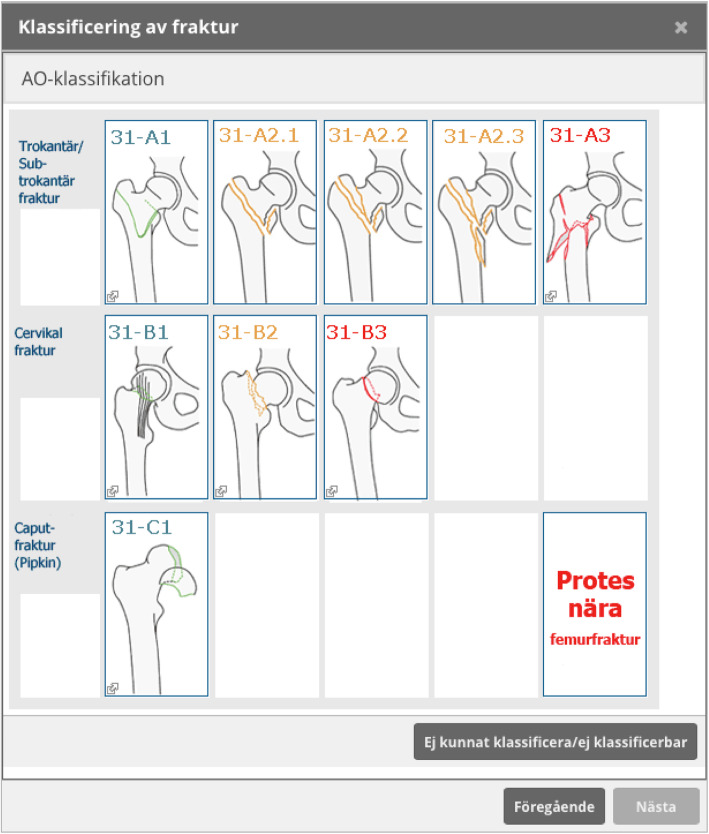
Classification of proximal femur fractures in the SFR

## Data Availability

The datasets used and/or analyzed during the current study are not publicly available. Data are available from the corresponding author on reasonable request.

## Abbreviations

FNF: Femoral neck fracture
bFNF: Basicervical femoral neck fracture
uFNF: Undisplaced femoral neck fracture
dFNF: Displaced femoral neck fracture
SFR: Swedish Fracture Register
IF: Internal fixation
SHD: Sliding hip device
IMN: Intramedullary nail
RCT: Randomized clinical trial
